# Prevalence and Impact of Academic Violence in Medical Education

**DOI:** 10.3390/ijerph191811519

**Published:** 2022-09-13

**Authors:** Patricia Costa Mincoff Barbanti, Sérgio Ricardo Lopes de Oliveira, Aline Edlaine de Medeiros, Mariá Românio Bitencourt, Silvia Veridiana Zamparoni Victorino, Marcos Rogério Bitencourt, Ana Carolina Jacinto Alarcão, Paulo Acácio Egger, Fernando Castilho Pelloso, Deise Helena Pelloso Borghesan, Makcileni Paranho de Souza, Vlaudimir Dias Marques, Sandra Marisa Pelloso, Maria Dalva de Barros Carvalho

**Affiliations:** 1 State University of Maringa, Maringá 87020-900, Brazil; 2 Centro Universitário Cesumar, Maringá 87050-900, Brazil; 3 Faculdade Adventista do Paraná, Maringá 87130-000, Brazil; 4 Fundação Estadual de Atenção Especializada à Saúde de Curitiba, Curitiba 81130-160, Brazil; 5 Centro Universitário Uningá, Maringá 87035-510, Brazil

**Keywords:** mistreatment, medical students, mental health, medical education, Brazil

## Abstract

Situations of mistreatment in the academic environment are prevalent worldwide, but research in this area is scarce in middle-low-income countries. This study aimed to estimate the prevalence of mistreatment inflicted against Brazilian medical students. In addition, characterize these situations and analyze their consequences. Cross-sectional study conducted with 831 medical students from public and private institutions. Absolute and relative frequencies of the analyzed variables and possible associations were determined through univariate and multivariate logistic regression. Chi-square test of association with second-order Rao-Scott adjustment was also used. The response rate was 56%. Public institution pointed to a higher prevalence of mistreatment when compared to private (59% versus 43%). Female students were the most affected. Verbal and psychological aggression was more prevalent. The aggressor usually was a faculty member. Mistreatment incidence increased over the years of training, with higher rates in the internship. About 94% of the students felt affected in anyway, with 77% feeling diminished and depressed. More than 50% reported impaired academic performance. Almost 30% sought help from experts. The reporting rate was extremely low. Adequate identification of the situations by the victims, safe reporting mechanisms and, an educational system capable of maintaining an appropriate learning environment are essential to break this destructive cycle.

## 1. Introduction

Situations of mistreatment in the academic environment include a wide range of behaviors that students find humiliating, hostile or abusive, such as harassment (verbal, psychological or sexual), discrimination, depreciation, and physical aggression. Such situations are prevalent worldwide among medical students and had been pointed out since the early 1980s [1]. Mistreatment had already been reported among undergraduate students and residents in many countries, with prevalence rates varying between 25 and 89%. The United States [2], Australia [3], New Zealand [4], Ireland [5], Brazil [6] and Japan [7] are some of these countries.

As already mentioned by Rautio et al. [8], mistreatment is not exclusive to medical training environments; however, when compared to courses in the areas of Humanities, Science and Technology, higher prevalence was found in medical courses. Even though this is a problem already exposed and discussed for about four decades, many studies continue to point to high frequencies [6,9,10].

These episodes can have profoundly negative effects on mental health, social life, learning and academic performance, as well as on patient care. Anxiety, depression, impairment in the relationship with faculty members, decrease in performance and even suicidal ideation have already been pointed out as consequences of a hostile learning environment [2,11,12]. Fleming & Smith [13] when investigating the impact of mistreatment on the professional career pointed out those such professionals are more likely to experience post-traumatic stress, depression, career dissatisfaction and suicide.

This research aimed to estimate the prevalence of situations of academic violence among Brazilian medical students and characterize such situations, identifying the different types of mistreatments perpetrated, the main aggressors, the frequency of episodes, the reporting rate and the impacts caused on these students.

In Brazil, as well as in other countries with medium/low income per capita, there are few studies regarding the occurrence and possible consequences of these different types of mistreatments during medical training. A systematic review on the topic, published in 2014, warned that only 6 of the 59 included studies had been carried out in middle/low-income countries [14]. In addition to this lack of studies, there is still the possibility of finding different panoramas on the subject if we consider the universe of public and private institutions in Brazil, given the organizational and administrative differences between them.

As pointed out in the Brazilian Institute of Geography and Statistics (IBGE) [15] datasets, Brazil is a country with continental proportions. It has 213.3 million inhabitants, of which only the southern region of the country has about 30.4 million inhabitants. So, national, and even regional research, demands high cost with logistics, time, and partnership between institutions, which can take years to occur. Meanwhile, academic violence may be occurring in our educational institutions, without those involved even being aware of this fact. This research represents a starting point to draw attention to the problem.

## 2. Methods

### 2.1. Study Design/Study Population/Data Collection

This research characterizes a cross-sectional study with a sample of medical students from the first to the sixth year of two universities located in southern Brazil, in which one of them was a public institution and the other was a private institution.

Students regularly enrolled in February 2020 were considered an eligible population (1228 students from the private university and 250 from the public). Of these, 709 students from the private and 122 from the public participated in the study. The disproportionate number of students in the private vs. public institution is due to the fact that the researched private university has 300 vacancies in the entrance exam per year since 2018, and the public one has only 40 vacancies for more than 10 years.

Data collection was performed using an online survey and was based on the publication by Barreto et al. [6]. An invitation containing the link (https://forms.gle/1YngZ7ADmzk7oWVH8, accessed on 12 April 2022) to participate in the research was sent via e-mail and on the WhatsApp application, and participation was voluntary after reading and agreeing with a free and informed consent form (FICF). Data collection was carried out between 17 February to 17 March 2020. Once the filling and sending of the questionnaire was completed, new accesses were blocked, thus avoiding multiple responses from the same participant.

To ensure a good percentage of respondents, weekly reminders were sent during the collection phase by e-mail and social network applications (WhatsApp and Facebook). All students who had not responded to the survey received the same number of reminders. In this way, everyone had the same opportunity to participate.

### 2.2. Ethical Considerations

An appropriate Human Research Ethics Committee (register nº 3.614.945 and 4.107.858) approved the project.

### 2.3. Data Collection Instrument

The collection instrument was composed of two main sections and elaborated using Google forms tool. In the first section, data related to the respondent’s sociodemographic and student characteristics were collected and, in the second section, data related to the situations of mistreatment experienced or not by the students.

The questionnaire addressed the following types of mistreatment/academic violence:

Verbal (scream/shout; depreciation/humiliation);

Psychological (assignment of tasks for punitive purposes; threat of injury; disparaging comments about career; harassment; racial/religious discrimination; threat of physical aggression);

Sexual (sexual harassment and discrimination);

Physical (slap/punch, push, kick).

When answering that they have not suffered any type of mistreatment described above, the questionnaire directed the respondents to complete filling and send the answers. When answering that they have already suffered an episode of mistreatment, the questionnaire directed the respondents to questions that characterize the situations (type, frequency, perpetrator, perceived severity, and effects caused by aggression). In the end, additional questions also assessed whether the episode was reported and to whom, and if not, what was the reason for the silence.

### 2.4. Data Analysis

#### Descriptive Analysis

Descriptive analysis was performed with calculation of the absolute and relative frequencies of the data found to characterize the research participants.

### 2.5. Association

To verify the existence of an association between the variables of interest and the institutions, the chi-square (χ^2^) test of association with second-order Rao-Scott adjustment was use, which allowed the analysis of associations for questions with more than one alternative [16]. For contingency tables with expected values lower than 5, Fisher’s exact test was used.

### 2.6. Logistic Regression

Through univariate logistic regression, possible associations between the characteristics of the students and the probability of having suffered some types of mistreatments were investigated by estimating the odds ratios (OR) as a measure of effect, with confidence interval of 95% and significance when *p* < 0.05. The variables that presented a significant association with the variable of interest were selected and included in the multivariate model, which in turn, estimated the adjusted odds ratio, considering possible interactions between such variables [17].

The quality of the adjustment of the multivariate model was assessed using the Hosmer and Lemeshow test, using the number of groups g = 10 [18]. At the end, the ROC curve was constructed to evaluate the discrimination of the proposed logistic model.

All analyses were performed using the statistical environment R (R Development Core Team, 2015) [19], version 3.5 (R Foundation for Statistical Computing, Vienna, Austria).

## 3. Results

### 3.1. Response Rate and Sociodemographic and Student Characterization of the Participants

Of the 1228 students from the private institution and 250 students from the public institution that were invited to participate, 709 (57.7%) and 122 (48.8%), respectively, accessed the survey link and fully completed the questionnaire. The sociodemographic and student characteristics of the participants are shown in Table 1.

Among the characteristics that showed to be significantly associated with the institutions, the female gender and the house-sharing stood out (*p* < 0.001). The private institution had a higher percentage of women enrolled (68.4% versus 50.8%) and students living alone (50.5% versus 18.0%) than the public.

The other variables evaluated did not show a significant association with the institution, indicating that the distribution of frequencies in both groups is close. About half of the sample was between 21 and 24 years old (47.3%) and was single (51.5%). They had predominantly white skin color/ethnicity (85.5%) and were distributed, equivalently, from the first to the sixth year of the course. Being satisfied with the professional choice was the option indicate by 92% of the participants, although more than 30% had already considered dropping out the course.

### 3.2. Situations Characterizing Mistreatment

**Figure 1:**
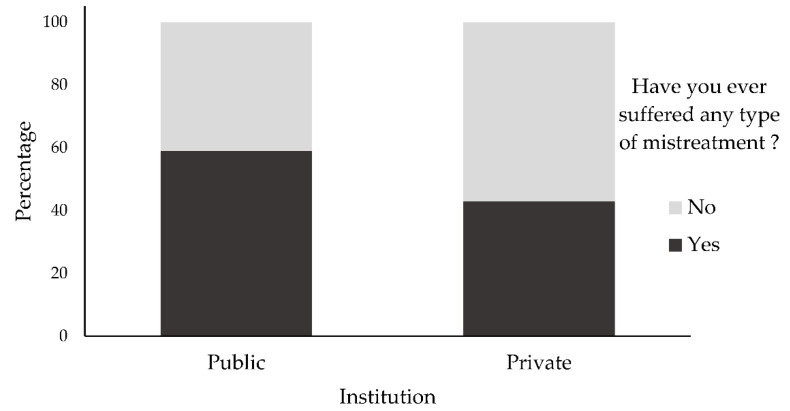
Frequency distribution of the responses of the research participants in relation to having already suffered some type of mistreatment, by institution. Source: Own elaboration.

About 59% participants from public institution and 43% from the private reported having suffered some type of mistreatment (Figure 1).

In both institutions, the mistreatment situations were mostly perpetrated by faculty (85.6%), followed by preceptor (27.4%), and doctors from the service where the students do internships (23.2%) (Table 2).

From Table 2, it was also possible to note that the most common types of mistreatments were verbal harassment—humiliation, depreciation or swearing (66.3%), and psychological aggression—negative comments about their future career (61.4 %), noting that the first one was more common among public institution students and the second, among private institution students (*p* = 0.034).

Regarding the impacts generated, the results obtained were also remarkably similar between the schools. Only 5.2% of the entire sample related that the aggressor’s attitude did not affect them in any way, while 94.8% felt affected. Feeling diminished, depressed, and stressed were commonly report. Academic performance was also negatively affected. Almost 30% had to seek for professional help from psychiatrists or psychologists to deal with the situation (Table 3).

Despite all this harm, the reporting rates were extremely low in both institutions (9.4%). Among the reasons given as justifications for non-notification, the main one was thinking that nothing would be done about it, which was reported by 72.1% of the participants (Table 4).

Other data to be emphasized and that are not shown in the tables are those involving the highest prevalence of mistreatment among internship students and those specifically involving sexual harassment/discrimination, in both institutions. It was observed that 65% of the students from the internship and 36% of the students from the 1st to the 4th year at private institution have already experienced some episode of mistreatment. Among students at public institution, the rate found was 82% at the internship and 48% at the 4 first years of the course, indicating that this rate practically doubles during the internship. It was also noticed that episodes involving sexual harassment/discrimination are far superior in this period of academic training.

### 3.3. Associations between the Students’ Characteristics and the Chances of Suffering Mistreatment

The variables that presented a significant association with the fact that the student had already suffered mistreatment in univariate logistic model (Supplementary Material Table S1) were then included in a multiple logistic model, allowing the calculation of the odds ratio estimates (adjusted OR), with their respective intervals of 95% confidence and *p*-values. The results obtained are described in Table 5.

The results obtained confirmed that studying in the private institution (*p* < 0.001), being male (*p* < 0.001) and be attending the 1st, 2nd, or 3rd year of the course (*p* < 0.001, *p* < 0.001 and *p* = 0.009, respectively), represented protective factors for mistreatment. Likewise, the age between 25–29 years old and having already thought about dropping out the course, remained as risk factors. It is quite likely that such situations of academic violence represent a frequent cause of thinking about giving up of the future professional career.

### 3.4. Analysis of the Quality of the Adjustment of the Multivariate Model

**Figure 2:**
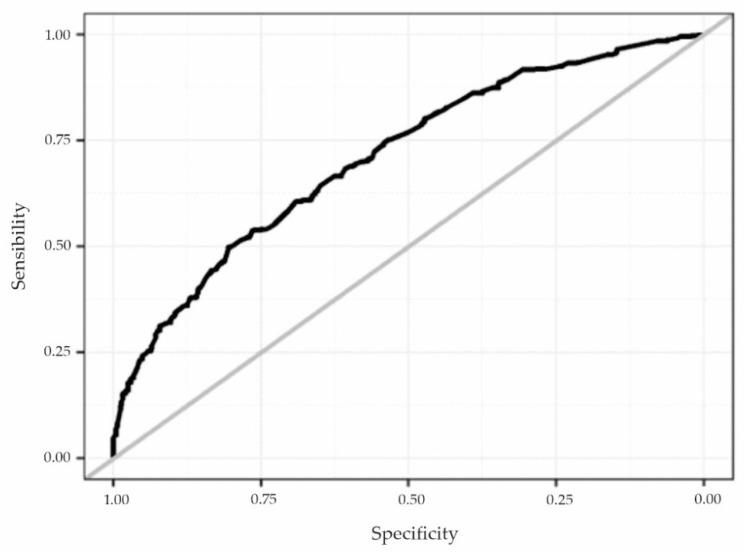
ROC curve of the multivariate logistic regression model used. Source: own elaboration.

Finally, the Hosmer and Lemeshow test showed that there was not enough evidence to indicate that the model was poorly adjusted (*p* = 0.2877), and the construction of the ROC curve (Figure 2) indicated a reasonable discriminatory power of the model, with area under the curve of 0.712.

These findings suggest that there may be other factors not explored, regarding mistreatment in the academic environment, other than those related to the victim’s characteristics.

## 4. Discussion

Although few studies on the subject had already been carried out in Brazil, we believe that this was the first that, in addition to evaluating mistreatment among medical students, aims to compare the reality between public and private institutions. The differences found may represent different starting points in the implementation of effective strategies to curb or, at least, minimize these situations.

In this study, the estimated prevalence of mistreatment was 59% at public institution and 43% at the private. As this was a cross-sectional study, with sample composed of volunteers, selection bias may have occurred. We may have had greater participation of the individuals mobilized by the subject, who had already gone through the situation described in the research title. It would result in overestimation of the prevalence found. On the other hand, we may have had a scenario where students who had already been exposed to such episodes, preferred not to participate and not to declare the mistreatment suffered by fear and insecurity. In this case, there would be a prevalence underestimation.

However, the prevalence found was slightly below that described in the meta-analysis developed by Fnais et al. [14]. When analyzing 30 studies also carried out with undergraduate medical students, they reached a combined prevalence of 59.6%. In addition, the use of online data collection strategy, in which the answers could be given outside the school environment, associated with guaranteed anonymity and confidentiality, facilitated the completion and declaration of situations of mistreatment.

Educational institutions, despite providing a space for education, socialization, and training of the individual, may be susceptible to inequality between peers (students, teachers, other professionals), which corroborates the mistreatment emergence, which can be seen as natural or be ignored and/or devalued [20]. Olasoji [21] pointed out that many educators consider complaints of violence in the study environment meaningless, and many others still consider some level of humiliation or severe criticism as necessary for the adequate training of the professionals. In this study, we detected that many students also come to believe that such situations are necessary to form a well-trained professional. About 20% of the victims believed that the episodes made them stronger and well prepared to deal with the typical situations of the medical profession.

The present research also reaffirmed the existence of a strongly hierarchical medical culture that permeates teaching and learning relationships and perpetuates situations of mistreatment as “rites of passage” [22]. We identified a much higher prevalence of mistreatment among internship students (73.5%), when compared to the students from the 1st to 4th grades (42%). At the internship, the hierarchy becomes extremely evident, with the presence of the 5th and 6th year “intern” students, residents, preceptors, and chiefs of internship. Recently, Lall et al. [23] published a survey where 45% of more than 7.600 residents said they had experienced mistreatment, and 2% said they had suicidal thoughts during medical training. Sacramento et al. [24], also revealed that among the different social groups, physicians in training are more vulnerable to developing anxiety and depression disorders.

Our findings also demonstrated that the female gender was the most attacked, having been considered a risk factor for mistreatment and that the faculty were the main perpetrators of the reported episodes. These findings were coincident with other several studies [25,26,27]. Qualitative research about the experiences of the gender climate in clinical training indicated that women described many situations where they were personally involved, while the men mainly talked about incidents they had heard about or had observed as bystanders [28]. Gender discrimination is also clear when approaching specialty choice. In a survey conducted by Samuriwo et al. [29], medical students reported that expressions such as “ladylike manner” and “the need to man up” are often highlighted during the clerkships in an attempt to maintain gender predominance in specific specialties.

The private institution targeted in this study, besides having a higher number of women enrolled (62%) than the public one (48%), also had a much more expressive percentage of students living alone, a factor of greater psychosocial vulnerability. However, we found a higher prevalence of mistreatment at public institution, suggesting that other factors may be associated and not only those related to victim characteristics. As a first approach, it should be noted that in Brazil, the scarcity of investments by the government might be making it difficult to carry out training with continuing education for faculty at public institution and making it impossible to implement policies and strategies to control inadequate situations in this environment. In addition, the student’s access to coordinators/directors, for a possible complaint, is much more difficult when compared to the student’s access at private institution. In the latter, the student also assumes the role of a consumer who pays for a certain service and the concern with having the student satisfied is constant. Inevitably, this responsibility ends up being transferred, even partially, to the faculty. These facts may represent possible justifications for the public university, often unconsciously and cyclically (as it was the type of training that the vast majority received and they are passing on, believing it to be the right way), to still maintain, in a greater proportion, inappropriate and unnecessary behaviors during medical training.

Another data that corroborated this hypothesis was that psychological mistreatment/violence, such as making negative comments about the student’s career in the future, was more common among students from the private institution, and verbal aggressions, with swearing and acts of humiliation and depreciation, were more common among the students from public. This demonstrates that aggressions at private institution tend to be a little more veiled than in the public institution, perhaps due to apprehension and fear that the faculty members of this institution have to be reprimanded and suffer more severe penalties from their superiors, because of their attitudes.

On the other hand, it was noticed that some of the data found were remarkably similar between the institutions. One of these data involves episodes of sexual harassment/discrimination. They did not represent the most prevalent type of mistreatment in any of the researched institutions, but some facts draw attention and concerns. In both institutions, the chances of suffering sexual harassment/discrimination practically triple in the internship; the frequency of occurrence was higher than the other types of mistreatments evaluated; it was committed against women in about 90% of the cases and it was the type that least pointed out a faculty member as the aggressor. The internship represents the period of the graduation in which the students have more contact with other professionals involved in their formation, which generates greater interaction with other possible aggressors (residents, preceptors, attending physicians, and nurses from the hospitals where they perform the internships; patients and family members), which could justify the higher rates.

Another relevant data showed that the attacked student responded similarly to the episode of mistreatment suffered, regardless of studying in a public or private school. The effects of mistreatment are quite worrying and had already been pointed out and discussed by several authors. Assari & Lankarani [26] and Pedrelli et al. [30] related that anxiety, depression, and suicidal behavior are the three biggest mental health problems among university students who had been exposed to violence. Feelings of low self-esteem and intense stress were also present in a study published by Peres et al. [27]. Owoaje et al. [12] observed that students who are victims of mistreatment are more dissatisfied with their professional choice and, more often, consider dropping out the course. Hu et al. [2], demonstrated a positive association between a hostile environment and the development of burnout and suicidal ideation among the attacked students.

The consequences do not end there, and students are not the only ones affected: there is increasing evidence that abuse and mistreatment within the care team lead to worse outcomes for patients. Riskin et al. [31] have already demonstrated that rude attitudes can have deleterious effect on medical teams’ performance. Diagnosis and intervention, information sharing, workload, help, and communication were also harmed.

Despite all these consequences and even when medical schools have mechanisms to facilitate anonymous reporting [32] and students recognize the reporting procedure, the number of complaints is small and permeated by barriers that can make it difficult to carry out [32,33,34]. The belief that the incidents are not significant enough to be report; the fear that the report could damage the student-teacher relationship; the fear of retaliation; and the discrediting of the institutions’ attitudes are some of these barriers [35]. According to Castillo-Angeles et al. [34], some students also think that the complaint could negatively interfere in their careers in the future. In this research, unfortunately, only 10% of the episodes of mistreatment were reported and we noted that the denouncement system in both institutions investigated was deficient. In the public university the implementation of policies that allow the denouncement and subsequent actions taken by the educational system, probably had not yet been discussed. Any student, among the few who made a complaint, reported satisfaction with the outcome.

Once we are conniving with mistreatment in medical training, we accept that this practice is normal and doctors in training begin to incorporate bad behavior in their professional daily life. Regardless of the institution, the damage caused to the students is real and similar, so academic violence urgently needs to be avoided.

## 5. Conclusions

A multifaceted strategy to try to curb these episodes in the academic environment is necessary. Adequate identification of the situations by the victims, safe reporting mechanisms and, an educational system capable of maintaining an appropriate learning environment are essential.

Furthermore, it was realized that when it comes to the factors involved in the performing of an episode of mistreatment, we should not seek only the specific characteristics and weaknesses of the victims. The aggressor becomes a key player in this relationship, requiring further studies to understand better all the factors that possibly influence this scenario.

The expansion of the range of private and public institutions investigated is also necessary in further research. The low number of participating universities does not allow a deep knowledge of the different regions of Brazil. However, since the chosen institutions, considered as references, revealed a high prevalence of violence, this study points to the need to investigate other places. As pointed out in the literature review, the number of works in this direction is small and violence can increase in places where people are socially more vulnerable.

## Figures and Tables

**Table 1:** Frequency distribution of the characteristics of the research participants, by institution.

Variable	General		Public		Private		*p* Value
	*n*	%	*n*	%	*n*	%	
Gender							<0.001 *
Female	547	65.8%	62	50.8%	485	68.4%	
Male	284	34.2%	60	49.2%	224	31.6%	
Age (years old)							0.471
17 to 20	208	25.0%	35	28.7%	173	24.4%	
21 to 24	393	47.3%	58	47.5%	335	47.2%	
25 to 29	189	22.8%	26	21.3%	163	23.0%	
≥30	41	4.9%	3	2.5%	38	5.4%	
Skin color/ethnicity							0.021 *
White	710	85.5%	97	79.5%	613	86.5%	
Brown	74	8.9%	16	13.1%	58	8.2%	
Yellow	35	4.2%	8	6.6%	27	3.8%	
Black	11	1.3%	0	0.00%	11	1.5%	
Other	1	0.1%	1	0.8%	0	0.0%	
Who do you live with							<0.001 *
Alone	380	45.7%	22	18.0%	358	50.5%	
With parentes	237	28.5%	82	67.2%	155	21.9%	
With another family member	85	10.3%	6	4.9%	79	11.1%	
With friends	82	9.9%	7	5.8%	75	10.6%	
With partner	41	4.9%	5	4.1%	36	5.1%	
Another option	6	0.7%	0	0.0%	6	0.8%	
Relationship status							0.378
Single	428	51.5%	59	48.4%	369	52.0%	
Dating without living together	346	41.6%	57	46.7%	289	40.8%	
Married or living together	57	6.9%	6	4.9%	51	7.2%	
Religion							0.009 *
Catholic	469	56.5%	67	54.9%	402	56.7%	
Evangelical	128	15.4%	20	16.4%	108	15.2%	
Atheist/Agnostic	107	12.9%	26	21.3%	81	11.4%	
Spiritist	66	7.9%	5	4.1%	61	8.6%	
Other	61	7.3%	4	3.3%	57	8.1%	
Year of the course							0.493
1st	132	15.9%	17	13.9%	115	16.2%	
2nd	188	22.6%	29	23.8%	159	22.4%	
3rd	157	18.9%	21	17.2%	136	19.2%	
4th	132	15.9%	18	14.8%	114	16.1%	
5th	123	14.8%	16	13.1%	107	15.1%	
6th	99	11.9%	21	17.2%	78	11.0%	
Are you satisfied with your professional choice?							0.847
No	4	0.5%	0	0.0%	4	0.6%	
Not sure yet	62	7.5%	10	8.2%	52	7.3%	
Yes	765	92.0%	112	91.8%	653	92.1%	
Have you ever thought about dropping out the course?							0.636
No	574	69.1%	87	71.3%	487	68.7%	
Yes	257	30.9%	35	28.7%	222	31.3%	

* *p*-value < 0.05. Source: own elaboration.

**Table 2:** Frequency distribution of the characteristics of the mistreatment suffered by the research participants, by institution.

Variable	General		Public		Private		*p* Value
	*n*	%	*n*	%	*n*	%	
Type of mistreatment/academic violence *							0.034 *
Verbal—humiliation, depreciation or swearing	254	66.3%	53	72.6%	201	64.6%	
Psychological—negative comments about their future career	235	61.4%	36	49.3%	199	64.0%	
Psychological—threat of harming grades/evaluations	192	50.1%	35	47.9%	157	50.5%	
Verbal—scream, shout	116	30.3%	28	38.4%	88	28.3%	
Psychological—tasks with punitive purpose	85	22.2%	22	30.1%	63	20.3%	
Psychological—threat of disapproval	61	15.9%	18	24.7%	43	13.8%	
Sexual—situations of harassment	54	14.1%	9	12.3%	45	14.5%	
Psychological—misappropriation of credit	44	11.5%	8	11.0%	36	11.6%	
Psychological—ethnic or religious prejudice	37	9.7%	11	15.1%	26	8.4%	
Sexual—sexual discrimination	21	5.5%	2	2.7%	19	6.1%	
Psychological—threat of physical aggression	2	0.5%	0	0.0%	2	0.7%	
Physical aggression	2	0.5%	1	1.4%	1	0.3%	
Frequency of mistreatment occurrence							0.079
Rarely (1/2 times)	200	52.2%	29	40.3%	171	55.0%	
Sometimes (3/4 times)	140	36.6%	33	45.8%	107	34.4%	
Often (5 or more times)	43	11.2%	10	13.9%	33	10.6%	
Aggressor *							0.13
Faculty member	328	85.6%	64	87.7%	264	84.9%	
Preceptor	105	27.4%	12	16.4%	93	29.9%	
Doctor of the service where the student interns	89	23.2%	23	31.5%	66	21.2%	
Nurse	61	15.9%	9	12.3%	52	16.7%	
Resident	62	16.2%	11	15.1%	51	16.4%	
Patient/family member/companion	32	8.4%	6	8.2%	26	8.4%	
Another health professional	19	5.0%	2	2.7%	17	5.5%	
Colleagues	13	3.4%	4	5.5%	9	2.9%	
Other	10	2.6%	1	1.4%	9	2.9%	

Note. * The question admits more than one answer. Source: own elaboration.

**Table 3:** Frequency distribution of data related to the impact of mistreatment, by institution.

Variable	General		Public		Private		*p* Value
	*n*	%	*n*	%	*n*	%	
Severity of the aggressor’s attitude							0.584
It didn’t affect me	20	5.2%	5	6.9%	15	4.8%	
Affected a little	164	42.8%	33	45.9%	131	42.1%	
Affected a lot	199	52.0%	34	47.2%	165	53.1%	
Effects caused *^,1^							0.622
I felt diminished and depressed	282	77.7%	53	79.1%	229	77.4%	
It created a feeling of contempt for the faculty and hindered the later relationship with him	264	72.7%	51	76.1%	213	72.0%	
It caused me intense stress	252	69.4%	46	68.7%	206	69.6%	
It created a poor learning environment, reflecting impairment in academic performance	186	51.2%	36	53.7%	150	50.7%	
It made me seek for professional help (psychiatrist or psychologist)	101	27.8%	13	19.4%	88	29.7%	
It made me study harder and made me stronger to face the typical situations of the medical profession	73	20.1%	17	25.4%	56	18.9%	
It made me increase my consumption of alcoholic beverages and/or other legal drugs	30	8.3%	4	6.0%	26	8.8%	

Note. * The question admits more than one answer. ^1^ Percentages calculated in relation to the respondents who pointed out that the aggressor’s attitude affected them in some way. Source: own elaboration.

**Table 4:** Frequency distribution of data related to reporting episodes, by institution.

Variable	General		Public		Private		*p* Value
	*n*	%	*n*	%	*n*	%	
Reported the fact to someone in the coordination of the course or direction of the Health Sciences Center							0.116
No	347	90.6%	69	95.8%	278	89.4%	
Yes	36	9.4%	3	4.2%	33	10.6%	
Why did you not report? *^,1^							0.477
For thinking that nothing would be done about it.	250	72.1%	53	76.8%	197	70.9%	
For fear of reprisal (related to grades and evaluations)	163	47.0%	37	53.6%	126	45.3%	
For thinking that I could handle/resolve the situation by myself.	117	33.7%	22	31.9%	95	34.2%	
Was in doubt if the fact really represented an inappropriate attitude on the part of the perpetuator or if it was part of the normal learning proccess of the course.	96	27.7%	16	23.2%	80	28.8%	
What did you think of the outcome of the complaint? ^2^							0.545
I felt dissatisfied; nothing was done in an attempt to help me	26	72.2%	3	100.0%	23	69.7%	
I felt satisfied with the attitude of the coordination/direction	10	27.8%	0	0.0%	10	30.3%	

Note. * The question admits more than one answer. ^1^ Percentages calculated in relation to respondents who did not report. ^2^ Percentages calculated in relation to respondents who reported. Source: own elaboration.

**Table 5:** Multivariate analysis of having already suffered mistreatment in function of the factors under study.

Variable	OR (Adjusted)	CI (95%)	*p*-Value
Institution			
Public	1	-	-
Private	0.45	0.29–0.68	<0.001 *
Gender			
Female	1	-	-
Male	0.5	0.36–0.69	<0.001 *
Age (years old)			
17 to 20	1	-	-
21 to 24	1.25	0.83–1.9	0.293
25 to 29	1.76	1.04–3	0.035 *
≥30	1.27	0.57–2.8	0.554
Relationship status			
Single	1	-	-
Dating without living together	1.22	0.89–1.66	0.214
Married or living together	0.93	0.5–1.72	0.807
Year of the course			
6th	1	-	-
5th	1.55	0.86–2.81	0.148
4th	0.67	0.38–1.18	0.166
3rd	0.47	0.26–0.82	0.009 *
2nd	0.36	0.2–0.65	<0.001 *
1st	0.24	0.12–0.45	<0.001 *
Have you ever thought about dropping out the course?			
No	1	-	-
Yes	1.5	1.09–2.07	0.014 *

* *p*-value < 0.05. Source: own elaboration.

## Supplementary Materials

The following supporting information can be downloaded at: https://www.mdpi.com/article/10.3390/ijerph191811519/s1, Table S1: Univariate analysis of having already suffered mistreatment in function of the factors under study and the result of the chi-square association test.
