# Supplementary material for: Prevalence and Impact of Academic Violence in Medical Education
Source: Int J Environ Res Public Health. 2022 Sep 13;19(18):11519. doi: 10.3390/ijerph191811519 (PMC9517415; doi:10.3390/ijerph191811519)
Supplement: Supplementary file 1 [file ijerph-19-11519-s001.zip › ijerph-1882496-supplementary.pdf]

Supplementary Material

Table S1-Univariate analysis of having already suffered mistreatment in function of the factors under study and the result of the chi-square association test.

Variable	Have you ever suffered mistretament No	Have you ever suffered mistretament Yes	OR (gross)	CI (95%)	*p*-value	χ^2 test *p*-value
Institution						0.003*
Public	50 (11%)	72 (19%)	1	-	-	
Private	398 (89%)	311 (81%)	0.54	0.37 - 0.8	0.002*	
Gender						0.007*
Female	276 (62%)	271 (71%)	1	-	-	
Male	172 (38%)	112 (29%)	0.66	0.49 - 0.89	0.006*	
Age (years old)						< 0.001*
17 to 20	145 (32%)	63 (16%)	1	-	-	
21 to 24	208 (46%)	185 (48%)	2.05	1.44 - 2.93	< 0.001*	
24 to 29	74 (17%)	115 (30%)	3.58	2.37 - 5.45	< 0.001*	
≥30	21 (5%)	20 (5%)	2.19	1.11 - 4.34	0.024*	
Skin color/ethnicity						0.335
White	384 (86%)	326 (85%)	1	-	-	
Brown	42 (9%)	32 (8%)	0.9	0.55 - 1.45	0.661	
Yellow	19 (4%)	16 (4%)	0.99	0.5 - 1.96	0.981	
Black	3 (1%)	8 (2%)	3.14	0.9 - 14.43	0.093	
Other	0 (0%)	1 (0%)	--	--	--	
Who do you live with						0.751
Alone	206 (46%)	174 (45%)	1	-	-	
With parentes	126 (28%)	111 (29%)	1.04	0.75 - 1.44	0.8	
With another family member	49 (11%)	36 (9%)	0.87	0.54 - 1.4	0.565	
With friends	46 (10%)	36 (9%)	0.93	0.57 - 1.5	0.756	
With partner	19 (4%)	22 (6%)	1.37	0.72 - 2.64	0.339	
Another option	2 (0%)	4 (1%)	2.37	0.46 - 17.23	0.323	
Relationship status						0.019*
Single	251 (56%)	177 (46%)	1	-	-	
Dating without living together	169 (38%)	177 (46%)	1.49	1.12 - 1.98	0.007*	
Married or living together	28 (6%)	29 (8%)	1.47	0.84 - 2.56	0.174	
Religion						0.308
Catholic	264 (59%)	205 (54%)	1	-	-	
Evangelical	67 (15%)	61 (16%)	1.17	0.79 - 1.74	0.426	
Atheist/Agnostic	56 (13%)	51 (13%)	1.17	0.77 - 1.79	0.458	
Spiritist	28 (6%)	38 (10%)	1.75	1.04 - 2.97	0.036*	
Other	33 (7%)	28 (7%)	1.09	0.64 - 1.87	0.746	
Year of the course						< 0.001*
1st	35 (8%)	64 (17%)	1	-	-	
2nd	35 (8%)	88 (23%)	1.37	0.78 - 2.43	0.272	
3rd	66 (15%)	66 (17%)	0.55	0.32 - 0.93	0.027*	
4th	92 (21%)	65 (17%)	0.39	0.23 - 0.65	< 0.001*	
5th	120 (27%)	68 (18%)	0.31	0.18 - 0.51	< 0.001*	
6th	100 (22%)	32 (8%)	0.18	0.1 - 0.31	< 0.001*	

Are you satisfied with your professional choice?						0.469
No	3 (1%)	1 (0%)	1	-	-	
Not sure yet	30 (7%)	32 (8%)	3.2	0.39 – 66.64	0.325	
Yes	415 (93%)	350 (91%)	2.53	0.32 - 51.28	0.422	
Have you ever thought about dropping out the course?						0.002*
No	331 (74%)	243 (63%)	1	-	-	
Yes	117 (26%)	140 (37%)	1.63	1.21 – 2.19	0.001*	
Total	**448 (100%)**	**383 (100%)**	-	-	-	-

* *p*-value < 0.05; OR: Odds Ratio; CI: Confidence Interval.

Figure S1-ROC curve of the multivariate logistic regression model used.

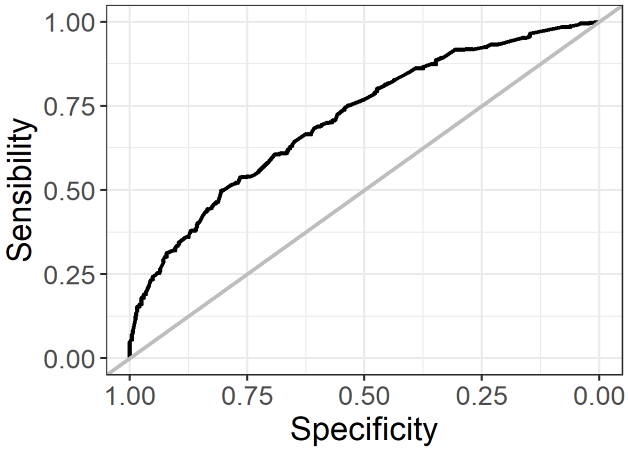

Source: Own elaboration
